# Hypofractionated radiation therapy and temozolomide in patients with glioblastoma and poor prognostic factors. A prospective, single-institution experience

**DOI:** 10.1371/journal.pone.0217881

**Published:** 2019-06-06

**Authors:** Paola Anna Jablonska, Ricardo Diez-Valle, Jaime Gállego Pérez-Larraya, Marta Moreno-Jiménez, Miguel Ángel Idoate, Leire Arbea, Sonia Tejada, Maria Reyes Garcia de Eulate, Luis Ramos, Javier Arbizu, Pablo Domínguez, José Javier Aristu

**Affiliations:** 1 Department of Radiation Oncology, Clínica Universidad de Navarra, Pamplona, Spain; 2 Department of Neurosurgery, Clínica Universidad de Navarra, Pamplona, Spain; 3 Department of Neurology, Clínica Universidad de Navarra, Pamplona, Spain; 4 Department of Anatomic Pathology, Clínica Universidad de Navarra, Pamplona, Spain; 5 Department of Radiology, Clínica Universidad de Navarra, Pamplona, Spain; 6 Department of Nuclear Medicine, Clínica Universidad de Navarra, Pamplona, Spain; University of South Alabama Mitchell Cancer Institute, UNITED STATES

## Abstract

**Background:**

Hypofractionated radiation therapy is a feasible and safe treatment option in elderly and frail patients with glioblastoma. The aim of this study was to evaluate the effectiveness of hypofractionated radiation therapy with concurrent temozolomide in terms of feasibility and disease control in primary glioblastoma patients with poor prognostic factors other than advanced age, such as post-surgical neurological complications, high tumor burden, unresectable or multifocal lesions, and potential low treatment compliance due to social factors or rapidly progressive disease.

**Material and methods:**

GTV included the surgical cavity plus disease visible in T1WI-MRI, FLAIR-MRI and in the MET-uptake. The CTV was defined as the GTV plus 1.5–2 cm margin; the PTV was the CTV+0.3 cm margin. Forty, fourty-five, and fifty grays in 15 fractions were prescribed to 95% of PTV, CTV, and GTV, respectively. Treatment was delivered using IMRT or the VMAT technique. Simultaneously, 75 mg/m^2^/day of temozolomide were administered.

**Results:**

Between January 2010 and November 2017, we treated a total of 17 patients. The median age at diagnosis was 68-years; median KPS was 50–70%. *MGMT*-methylation status was negative in 5 patients, and 8 patients were *IDH*-wildtype. Eight of 18 patients were younger than 65-years. Median tumor volume was 26.95cc; median PTV volume was 322cc. Four lesions were unresectable; 6 patients underwent complete surgical resection. Median residual volume was 1.14cc. Progression-free survival was 60% at 6 months, 33% at 1-year and 13% at 2-years (median OS = 7 months). No acute grade 3–5 toxicities were documented. Symptomatic grade 3 radiation necrosis was observed in one patient.

**Conclusions:**

Patients with poor clinical factors other than advanced age can be selected for hypofractionated radiotherapy. The OS and PFS rates obtained in our series are similar to those in patients treated with standard fractionation, assuring good treatment adherence, low rates of toxicity and probable improved cost-effectiveness.

## Introduction

Glioblastoma multiforme (GB) is the most common primary brain tumor. It usually develops in the sixth decade of life, with the median age at diagnosis of 64 years [1]. The standard of care consists of surgical resection, followed by conventional fractionated radiation therapy (CFRT) with concurrent and adjuvant temozolomide (TMZ). This approach shows a median overall survival (OS) time of 14.6 months (13.2–16.8 months) and a 5-year overall survival (OS) rate of 9.8% (6.4–14.0%) [2]. The impact of hypofractionated radiation therapy (HFRT) has recently been investigated. Results of initial research aimed at reducing the overall treatment time in frail and elderly GB patients suggest using both TMZ and HFRT as standard treatment option in this subgroup of patients [3].

However, there is little data to support using HFRT/TMZ in GB patients other than the elderly and fragile, such as those with other conditions conferring a poor prognosis. The purpose of our study was to determine whether HFRT with simultaneous and adjuvant TMZ was feasible and could provide adequate disease control in primary GB patients with poor prognostic factors such as high tumor burden, unresectable or multifocal lesions, low Karnofsky Performance Status (KPS), presence of comorbidities, and/or unfavorable social factors.

## Materials and methods

All patients provided a written informed consent to the treatment and the use of their data for scientific purposes. The University of Navarra’s Ethics Committee approved the study. Patients with primary GB, 18 years of age or older, meeting any of the inclusion criteria (Table 1) were enrolled to receive HFRT and concomitant TMZ.

**Table 1:** Inclusion and exclusion criteria.

Inclusion Criteria	Exclusion Criteria
Tumor-related:	Brain stem tumors
biopsy or subtotal resection	KPS <50%
tumor volume >25cc	
multifocal tumors	
Surgery-related complications	
Low KPS/RPA score and patients’s comorbidity	
Rapidly progressive disease	
Unfavorable social/family factors	

Patients with brainstem tumors and a KPS <50% were excluded from the study. All patients underwent debulking surgery; in case of tumor unresectability, a biopsy procedure was performed in order to obtain tumor diagnosis. Subtotal resection was defined as less than 100% and more that 50% of the tumor. iPlan RT Treatment Planning Software version 5.0 was used to estimate the tumor volume at diagnosis and the residual tumor volume after surgical resection or biopsy. *MGMT* promoter methylation and *IDH* status were determined performing methylation specific polymerase chain reaction and immunohistochemistry staining, respectively. The radiation treatment planning involved image fusion of the brain 11C-Methionine-PET-CT-scan, multiparametric MRI using T1-weighted images and T2-FLAIR sequences. Residual disease in the PET study was defined using the maximum and mean T/N ratio values. All patients were immobilized with a thermoplastic mask and an individualized dental mold. The GTV was delineated as the entire surgical cavity plus eventual residual disease shown by T1WI contrast enhancement, T2-weighted FLAIR MRI and/or 11C-Methionine PET uptake. The CTV corresponded to the GTV plus a 1.5–2 cm margin, and the PTV included the CTV with 0.3 cm isotropic margin. HFRT was delivered within 4–6 weeks after the surgery or within 1–2 weeks after a biopsy, using either IMRT of VMAT techniques. The total dose prescribed was 40 Gy, 45 Gy, and 50 Gy in 15 daily fractions to the 95% of the PTV, CTV, and GTV, respectively.

All patients received concurrent oral TMZ (75 mg/m^2^ once daily) during the radiation therapy period, including the weekends. Adjuvant TMZ was prescribed one month after the end of HFRT, at 150–200 mg/m^2^ orally, once daily, for 5 consecutive days every 28 days up to 6–12 cycles or until disease progression. Antiepileptic drugs were prescribed only in patients with a history of at least one seizure. A database was created using IBM SPSS Statistics 20 software. The primary endpoints of the study were: OS, progression free survival (PFS), and treatment-related acute and chronic toxicities. Secondary endpoint was the evaluation of treatment compliance. Clinical outcome was evaluated by MRI one month after HFRT/TMZ and every 2–3 months thereafter. A minimum follow-up of 6 months was assured. Progressive disease was defined as radiological progression. Response evaluation was documented according to Response Assessment in Neuro-Oncology (RANO) working group [4]. If brain imaging could not be performed, symptomatic progression needing increased administration of corticosteroids was registered as progression of the disease. In case of doubt between progressive disease and radiation necrosis (RN), perfusion MRI and 11C-METPET were carried out. The toxicity was measured according to the CTCAE version 4.0.

## Results

### Patients and treatments

From January 2010 to November 2017, 17 patients with a primary GB diagnosis who met the inclusion criteria were enrolled. The median follow-up time for the whole cohort was 52 months (6–83). Patients and tumor characteristics are listed in Tables 1 and 2. In brief, the median age at diagnosis was 68 years (50–77). Eight (47%) patients were younger than 65 years old. Also, eight patients (47%) underwent complete tumor resection; 5 (29%) had a subtotal resection, and biopsy was performed in 4 patients (24%). Peri-lesional edema due to mass effect was observed in 7 patients (41%) at the time of diagnosis. The median PTV volume calculated was 321.75 cc (162.00–561.34) with a median PTV dose of 43.49 Gy (40.29–46.54), as shown in Table 3. As poor prognosis indicators, almost 50% of tumor biopsies were IDH *wild-type* and more than 50% of the tumors were unresectable or underwent incomplete surgery, with bulky characteristics on the RM imaging and 7 out of 10 lesions being bifocal or involving 3 or more foci. All patients completed the radiation treatment of HFRT with concurrent TMZ. Adjuvant TMZ was administered in 17 (100%) patients one month after the end of HFRT/TMZ.

**Table 2:** Patients’ characteristics.

Number of patients	17
**Gender:**	
Male	8
Female	9
**Age:**	
Median (range years)	68 (50–77)
<60	1
60–70	11
>70	5
**KPS:**	
<50%	0
50–70%	12
>70%	5
**RPA:**	
IV	4
V	11
VI	2

**Table 3:** Tumor characteristics.

**Median tumor size**	4.5cm (2.7–8 cm)
**Median tumor volume**	26.95cc (3.56–97.65cc)
**Poor molecular factors**	
*IDH* wild type	8 (47%)
*MGMT* unmethylated	5 (29%)
Median residual tumor volume	1.14cc (0.00–69.11)
**Mass effect**	
Yes	7
No	10
**Surgery**	
Complete	8 (47%)
Incomplete	5 (29%)
Unresectable	4 (24%)
**Median T/N ratio PET before HFRT**	
Mean T/N ratio	2.63 (1.23–6.53)
Max. T/N ratio	3.00 (1.23–9.56)
**Multifocal tumors**	
Unifocal	10
Bifocal	5
≥3 foci	2
**Corticoids after treatment**	
Yes	4 (24%)
No	13 (76%)

### Prognostic factor analyses

The conditions conferring worse patients’ prognosis according to the inclusion criteria are listed in Table 4.

**Table 4:** Conditions conferring worse patient prognosis.

	Number of Patients
**Tumor related**	
Tumor volume > 25cc	9
Subtotal resection	4
Biopsy	5
Two or more tumor foci, > 2 lobes involvement	7
IDH wild type	8
MGMT unmethylated	5
**KPS ≤60%**	5
**RPA score V-VI**	13
**Surgery related**	
Postsurgical meningitis	1
Postoperative intracranial abscess	1
Perioperative cerebral infarction	1
Nosocomial pneumonia	1
**Patient comorbidities**	
Previous history of cancer	1
Secondary neoplasm in active treatment	1
Advanced Lyme disease	1
Chronic idiophatic axonal sensorimotor PN	1
Chronic immunosupression	1
**Rapidly progressive disease**	2
**Social and family factors**	
Foreign patients	3
Lack of primary caregiver	1

### PFS and OS analyses

**Fig 1:**
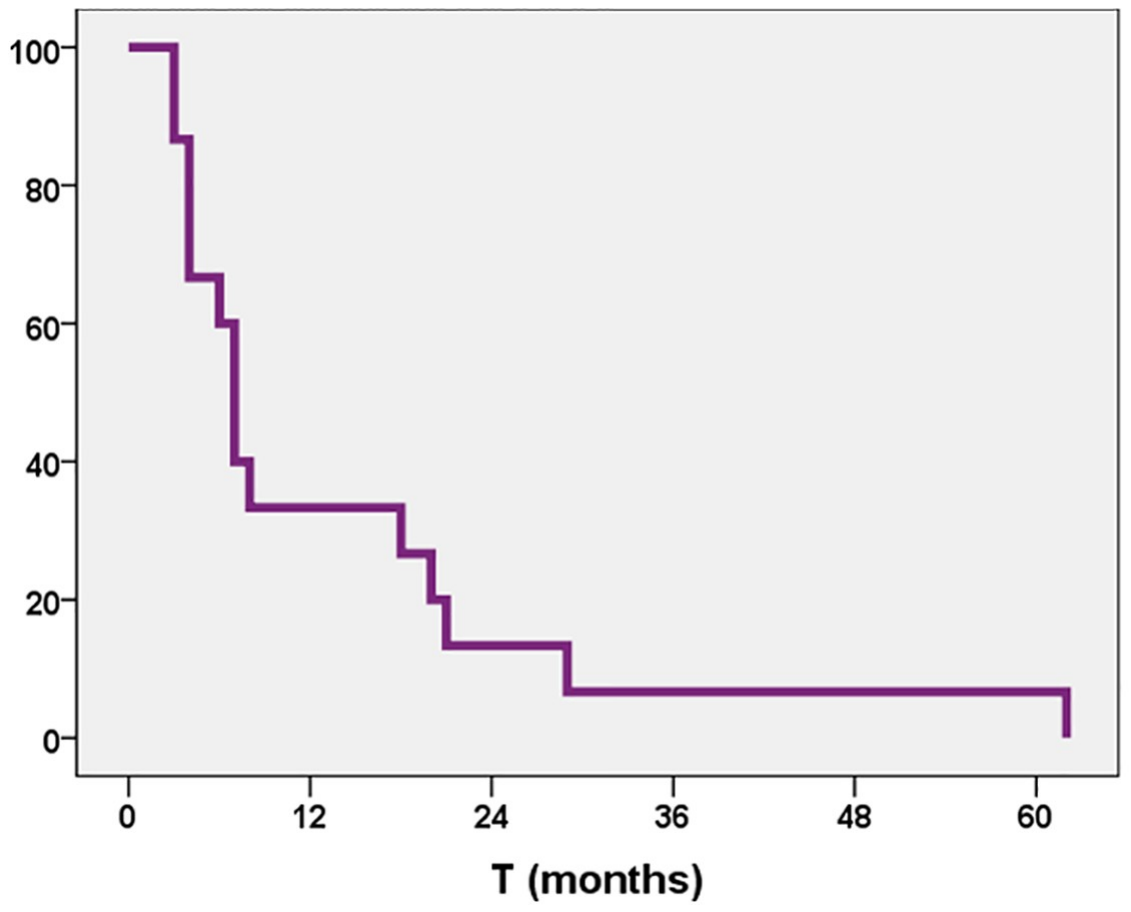
Overall survival rate.

**Fig 2:**
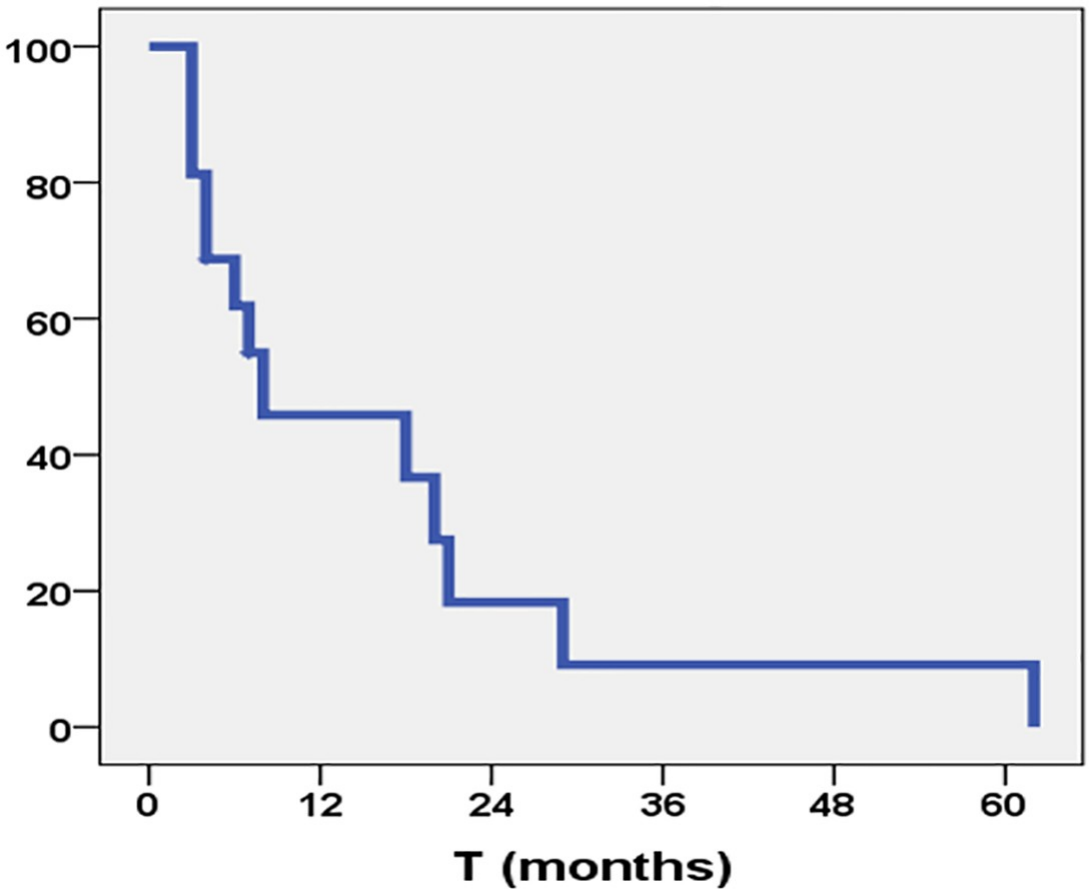
Progression-free survival rate.

The median OS time was 7 months; the 6-month, 1-, and 2-year OS rates were 62%, 46%, and 18%, respectively (Fig 1). At the last follow-up time, 3 (18%) patients were alive with disease, and 14 (82%) had died. The median PFS time was 7 months (95% CI 5.76–8.24), with 6-month, 1-, and 2-year PFS rates of 60%, 33%, and 13%, respectively (Fig 2). No patient was alive at 5 years after the time of diagnosis.

### Patterns of recurrence

Local recurrence was observed in 12 patients (71%). Distal failure was recorded in two patients (12%). Three patients (17%) showed clinical progression.

### Treatment toxicity

**Fig 3:**
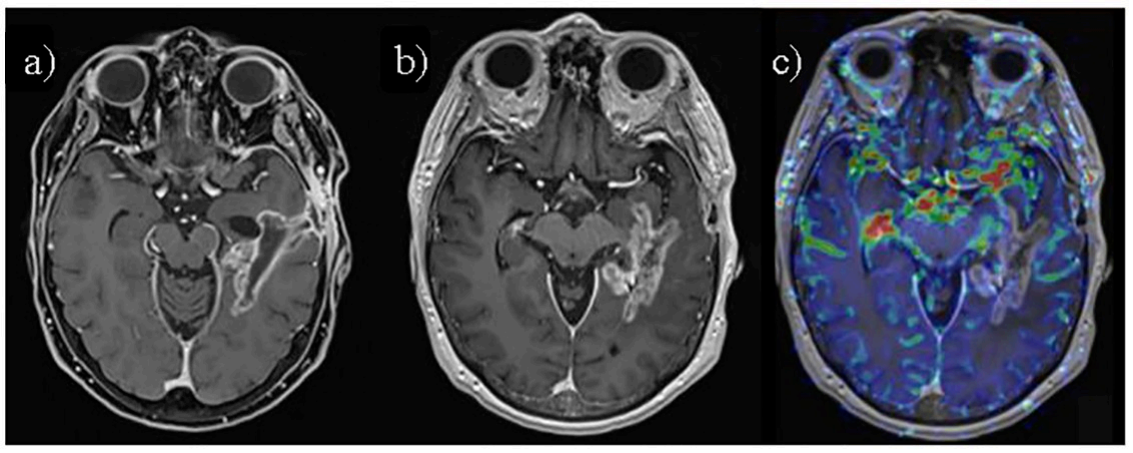
Symptomatic grade 3 radiation necrosis. a) Axial fat-saturated contrast-enhanced T1-weighted image (CE T1-WI) MRI 4 months after HFRT/TMZ, showing mostly linear enhancement surrounding the surgical cavity in left temporal lobe. b) Axial CE T1-WI of the same patient 2 months later (6 months after HFRT/TMZ), shows increased enhancement with a "soap-bubble" pattern, suggestive of radiation necrosis/pseudoprogression. c) Axial perfusion-weighted cerebral brain volume (CBV) map fused over CE T1-WI shows decreased perfusion values in the enhancing area, further supporting radiation necrosis.

Treatment-related acute grade 2 toxicity was observed in 3 patients (Table 5). One patient required active corticoid treatment. No acute grade 3–5 toxicities were observed. Symptomatic grade 3 radiation necrosis was observed in one patient at 6 months after them HFRT/TMZ, requiring treatment with bevacizumab (Fig 3). One patient presented with a subdural hygroma requiring surgical evacuation.

**Table 5:** Toxicity reported.

ACUTE			CHRONIC		
GRADE 2			GRADE 2		
Perilesional brain edema	1	6%	Asymptomatic brain edema	1	6%
Hematologic toxicity	1	6%	**GRADE 3**		
Anorexia and asthenia	1	6%	Symptomatic radiation necrosis	1	6%

## Discussion

To our knowledge, there is no data available on HFRT/TMZ in GB patients with poor prognostic factors other than advanced age or fragility. Our study shows that short course RT of 40 Gy in 15 fractions with concurrent TMZ is a feasible and safe treatment option for GB patients with unfavorable general conditions.

Age and performance status are the most relevant clinical prognostic factors for patients with malignant glioma [5]. The aggressiveness of this tumor can often be seen in routine clinical practice, as up to 10% of GB patients discontinue the treatment due to rapid disease progression [6]. In this sense, the design of therapeutic strategies that aim at shortening the overall treatment time without decreasing efficacy or increasing toxicity might be helpful. The data published have shown that short course RT and CFRT are equivalent in terms of outcome and safety [7]. These results have led to an increased use of hypofractionated RT schemes to improve quality of life for elderly and fragile patients.

A French study has reported that, in GB patients aged 70 years or more, the addition of RT to supportive care prolongs survival without compromising the quality of life or cognitive function [8]. However, the optimal dose in this subpopulation remains unclear. In 1994, Bauman et al. showed that elderly GB patients with a low pretreatment KPS (<50) might be adequately treated with a short, palliative course RT of 30 Gy in 10 fractions and that in patients ≥ 65 years old who remain fit (KPS >60), a higher dose RT could be attempted to provide a benefit in survival [9]. A phase III trial conducted by Roa and colleagues compared two different hypofractionation schemes (40 G y in 15 fractions and 25 Gy in 5 fractions) without concurrent TMZ in patients ≥ 65 years of age with KPS >50 [10]. No differences in OS, PFS, or quality of life were observed between the two arms. However, this study has been criticized for having a low statistical power and for the presence of other factors such as differences in patients’ characteristics, trial design, and treatment delivery that could have influenced the clinical outcomes. On the other hand, the Nordic Clinical Brain Tumor Study conducted by Malmström et al. reported poorer OS outcomes in GB patients ≥ 70 years old who were treated with CFRT (60 Gy in 30 fractions) in comparison to those who received a hypofractionated scheme of 34 Gy in 10 fractions [11]. The inferior survival in the group treated with CFRT could be partially explained by early treatment discontinuation because of low compliance in a substantial number of patients. Based on these results, HFRT might be suggested as a standard treatment in this scenario, although concurrent TMZ was not included in this study was lacking. In this respect, a retrospective study from Dana-Farber/Brigham and Women’s Cancer Center investigated the delivery of HFRT versus CFRT with or without temozolomide for older glioblastoma patients. After a propensity score adjustment, the authors observed that patients receiving HFRT with temozolomide had similar OS rates compared to those of patients receiving CFRT and TMZ [12]. However, no randomized trial has been conducted to directly compare these two regimens.

As described above, most of the research on HFRT has been conducted in elderly and fragile GB patients in anticipation of lower tolerance and poorer adherence using a conventional regimen of 60 Gy in 30 fractions in this group. After analyzing these data, we identified a prognostically similar subgroup that we believed could benefit from a hypofractionated treatment, especially in terms of higher compliance rates and quality of life. We decided to define this subgroup as all-age GB patients with disadvantageous radiological and molecular tumor characteristics, presence of other comorbidities, or neurological sequelae after the tumor surgery or diagnostic biopsy. We also identified patients with extraordinary rapidly progressive disease at the time of MRI and PET studies used for the radiation planning, shortly after the surgery, or after the diagnostic biopsy. Finally, we considered limited family support and social difficulties as negatively contributing factors to a full treatment adherence.

In our every-day clinical practice, we encounter many GB patients with such a poor pretreatment status, due to not only advanced aged or fragility. Therefore, based on preliminary studies, we contemplated the use of HFRT of 40 Gy in 15 fractions with concurrent Temozolomide as a valid treatment option in this setting. The results observed in our study have demonstrated an excellent compliance, as all patients were able to complete the radiation treatment. In addition, the toxicities observed were low, foreseeable, and easily manageable in outpatient treatment and at closer follow-up. The PFS and OS rates were comparable to those reported in current published data of series including patients with similar prognoses who were treated with CFRT [13]. Our results still need to be confirmed in a prospective trial with an appropriate design and a larger number of patients.

We believed it was important to take into account not only the clinical conditions of the GB patients, but also the morphological and molecular factors related to the tumor. It is well known that these glioblastoma-related factors have an impact on the course of the disease and hence the patient’s prognosis. It is also of note that our sample included GB patients older than 70 who had a poor KPS. According to the current NCCN Guidelines (Version 1.2018), patients with primary GB diagnosis, poor performance status (KPS <60), and age >70 should be treated with either temozolomide or be offered the best supportive care. Our data suggest that the use of HFRT could also be contemplated in selected patients with these characteristics. However, a direct comparison between BSC and HFRT would need to be conducted to evaluate the benefit of HFRT/TMZ in terms of PFS and quality of life.

Some studies in elderly patients with glioblastoma tried to determine whether the addition of TMZ to HFRT was more advantageous than HFRT alone. In a Phase III trial, Perry et al. concluded that HFRT plus TMZ in elderly newly diagnosed GB patients resulted in a longer PFS and OS than HFRT alone. In subgroup analysis, the median OS was nearly double in patients with *MGMT* methylated tumors, although the benefit was also found in patients with unmethylated *MGMT* tumors [14]. It is unknown whether TMZ acts purely as a radiosensitizer or not. Some authors suggest, that TMZ only adds cytotoxicity in combination with radiation, with no increase in cell sensitivity [15]. Other studies argue that TMZ is a powerful radiosensitising agent, enhancing the radiation response independently of the epigenetically silenced *MGMT* gene [16]. If that were truly the case, a lower amount of the total dose during hypofractionation could improve the toxicity profiles associated with TMZ, which is of significant importance in these poor prognosis groups of patients. In our study, conventionally fractionated, biologically effective doses (BED) prescribed to the 95% of the GTV, CTV, and PTV were equivalent to 66.83 Gy, 58.50 Gy, and 49.14 Gy, respectively. These doses were lower than the BED administered using the standard 60 Gy in 30 fractions scheme (BED equivalence of 72 Gy). We therefore can assume that our treatment results have been influenced by the radiosensitising effect of concomitant TMZ. For the future studies, hypofractionated regimens with concomitant TMZ and a higher BED could be attempted, despite of the poor pretreatment status of this group of patients, with an aim of improving the overall outcome.

Additionally, the possibility of using HFRT to treat patients between the ages of 16 and 65 years with newly diagnosed GB has been analyzed in a randomized phase II study in New Delhi, India [17]. Eighty-nine patients were randomized to CFRT (60 Gy in 30 fractions of 2 Gy) or HFRT (60 Gy in 20 fractions to high-risk PTV and 50 Gy in 20 fractions to low-risk PTV). Median OS in the CFRT and HFRT arms were 18.07 and 25.18 months, respectively (p value = 0.3). Only one patient in the HFRT arm required treatment interruption, and all patients completed the planned course of radiation. Toxicity was low, and no significant steroid dependency or other neurological toxicities were observed. Authors concluded that HFRT was comparable to CFRT in terms of survival outcome, with acceptable adverse effects. We believe that this will be the main subject of the future research on this matter, and we have initiated a phase II study that uses 64 Gy in 20 fractions prescribed to the high risk PTV defined by T1WI contrast enhancement and Methionine-PET-CT-scan positive areas.

As the main aim of this study was to test the feasibility and disease control rate of the 40 Gy in 15 fractions hypofractionated scheme, we have not recruited a control group, but our results are comparable to the previous clinical data available. The median OS of 7 months is similar to the median OS rate of 6.4 months reported by Roa [10] when using the same fractionation, as it is for the 2-year PFS rate of 11,2% shown with standard fractionation in the Stupp study [2] in comparison to 13% of 2-year PFS observed during the follow-up of our analysis.

## Conclusions

Our results show that HFRT with concurrent TMZ is a feasible therapeutic approach in patients with primary GB and other poor prognostic factors, assuring high treatment compliance and low toxicity rates. Dose escalation and reduction in overall treatment time are clear advantages of HFRT, while at least the same survival rates as a longer course of RT are maintained. However, our results are preliminary and non-comparative. More solid research is needed to define more robust selection criteria for HFRT beyond the indication for elderly and fragile patients before HFRT can be established as the new standard of care in newly diagnosed GB.

## Supporting information

S1 File Dataset. (XLSX)

## References

[1] Thakkar JP, Dolecek TA, Horbinski C, Ostrom QT, Lightner DD, Barnholtz-Sloan JS, et al Epidemiologic and Molecular Prognostic Review of Glioblastoma. Cancer epidemiology, biomarkers & prevention: a publication of the American Association for Cancer Research, cosponsored by the American Society of Preventive Oncology 2014; 23:10: 707–1996. 10.1158/1055-9965.EPI-14-0275 PMC4185005 25053711

[2] Stupp R, Hegi ME, van den Bent MJ, Taphoorn MJ, Janzer RC, Ludwin SK, et al Effects of radiotherapy with concomitant and adjuvant temozolomide versus radiotherapy alone on survival in glioblastoma in a randomised phase III study: 5-year analysis of the EORTC-NCIC trial. Lancet Oncol 2009; 10:459–466. 10.1016/S1470-2045(09)70025-7 19269895

[3] Wick W, Platten P, Meisner C, Felsberg J, Tabatabai G, Simon M, et al. Temozolomide chemotherapy alone versus radiotherapy alone for malignant astrocytoma in the elderly: the NOA-08 randomised, phase 3 trial. Lancet Oncol 2012; 13 (7): 707–715. 10.1016/S1470-2045(12)70164-X 22578793

[4] Wen PY, Macdonald DR, Reardon DA, Cloughesy TF, Sorensen AG, Galanis E, et al Updated Response Assessment Criteria for High-Grade Gliomas: Response Assessment in Neuro-Oncology Working Group. J Clin Oncol 2010; 28(11): 1963–1972. 10.1200/JCO.2009.26.3541 20231676

[5] Curran WJ Jr, Scott CB, Horton J, Nelson JS, Weinstein AS, Fischbach AJ, et al Recursive partitioning analysis of prognostic factors in three Radiation Therapy Oncology Group malignant glioma trials. J Natl Cancer Inst. 1993 5 5; 85(9): 704–710. 10.1093/jnci/85.9.704 8478956

[6] Zhou X, Liao X, Zhang B, He H, Shui Y, Xu W, et al Recurrence Patterns in Patients with High-Grade Glioma Following Temozolomide-Based Chemoradiotherapy. Molecular and Clinical Oncology 2016; 5(2): 289–294. 10.3892/mco.2016.936 27446566 PMC4950878

[7] Navarria P, Pessina F, Franzese C, Tomatis S, Perrino M, Cozzi L, et al Hypofractionated radiation therapy (HFRT) versus conventional fractionated radiation therapy (CRT) for newly diagnosed glioblastoma patients. A propensity score matched analysis. Radiother Oncol 2018; 127(1): 108–113. 10.1016/j.radonc.2017.12.006 29291951

[8] Keime-Guibert F, Chinot O, Taillandier L, Cartalat-Carel S, Frenay M, Kantor G, et al Radiotherapy for glioblastoma in the elderly. N Engl J Med 2007; 356: 1527–1535. 10.1056/NEJMoa065901 17429084

[9] Bauman S, Gaspar LE, Fisher BF, Halperin EC, Macdonald DR, Cairncross JG. A prospective study of short-course radiotherapy in poor prognosis glioblastoma multiforme. Int J Radiat Oncol Biol Phys 1994; 29: 835–839. 8040031 10.1016/0360-3016(94)90573-8

[10] Roa W, Kepka L, Kumar N, Sinaika V, Matiello J, Lomidze D, et al International Atomic Energy Agency Randomized Phase III Study of Radiation Therapy in Elderly and/or Frail Patients With Newly Diagnosed Glioblastoma Multiforme. J Clini Oncol 2015; 33(35): 4145–4150. 10.1200/JCO.2015.62.6606 26392096

[11] Malmström A, Grønberg BH, Marosi C, Stupp R, Frappaz D, Schultz H, et al Nordic Clinical Brain Tumour Study Temozolomide versus standard 6-week radiotherapy versus hypofractionated radiotherapy in patients older than 60 years with glioblastoma: the Nordic randomised, phase 3 trial. Lancet Oncol 2012; 13 (9): 916–926. 22877848 10.1016/S1470-2045(12)70265-6

[12] Arvold ND, Tanguturi SK, Aizer AA, Wen PY, Reardon DA, Lee EQ, et al Hypofractionated versus standard radiation therapy with or without temozolomide for older glioblastoma patients. Int J Radiat Oncol Biol Phys 2015: 92: 384–389. 10.1016/j.ijrobp.2015.01.017 25841623

[13] Stupp R, Mason WP, van den Bent MJ, Weller M, Fisher B, Taphoorn MJ, et al Radiotherapy plus concomitant and adjuvant temozolomide for glioblastoma. N Engl J Med 2005; 352(10):987–96. 10.1056/NEJMoa043330 15758009

[14] Perry JR, Laperriere N, O’Callaghan CJ, Brandes AA, Menten J, Phillips C, et al Short-course radiation plus temozolomide in elderly patients with glioblastoma. N Engl J Med 2017: 376: 1027–1037. 10.1056/NEJMoa1611977 28296618

[15] Chalmers AJ, Ruff EM, Martindale C, Lovegrove N, Short SC. Cytotoxic effects of temozolomide and radiation are additive- and schedule-dependent. Int J Radiat Oncol Biol Phys 2009; 75(5):1511–9. 10.1016/j.ijrobp.2009.07.1703 19931733

[16] van Nifterik KA, van den Berg J, Stalpers LJ, Lafleur MV, Leenstra S, Slotman BJ, et al Differential radiosensitizing potential of temozolomide in MGMT promoter methylated glioblastoma multiforme cell lines. Int J Radiat Oncol Biol Phys 2007; 11 15;69(4):1246–53. 10.1016/j.ijrobp.2007.07.2366 17967314

[17] Mallick S, Kunhiparambath H, Gupta S, Benson R, Sharma S, Laviraj Ma, et al Hypofractionated accelerated radiotherapy (HART) with concurrent and adjuvant temozolomide in newly diagnosed glioblastoma: a phase II randomized trial (HART-GBM trial). J Neurooncol. 2018 10;140(1):75–82. Epub 2018 Jun 23. 10.1007/s11060-018-2932-3 29936695

