# Correction: Hypofractionated radiation therapy and temozolomide in patients with glioblastoma and poor prognostic factors. A prospective, single-institution experience

**DOI:** 10.1371/journal.pone.0219614

**Published:** 2019-07-08

**Authors:** Paola Anna Jablonska, Ricardo Diez-Valle, Jaime Gállego Pérez-Larraya, Marta Moreno-Jiménez, Miguel Ángel Idoate, Leire Arbea, Sonia Tejada, Maria Reyes Garcia de Eulate, Luis Ramos, Javier Arbizu, Pablo Domínguez, José Javier Aristu

There is an error in the XML of the article. As a result, the third author’s name is listed in the citation incorrectly. The correct citation is: Jablonska PA, Diez-Valle R, Gállego Pérez-Larraya J, Moreno-Jiménez M, Idoate MÁ, Arbea L, et al. (2019) Hypofractionated radiation therapy and temozolomide in patients with glioblastoma and poor prognostic factors. A prospective, single-institution experience. PLoS ONE 14(6): e0217881. https://doi.org/10.1371/journal.pone.0217881
